# Synergistic effects of ISL1 and KDM6B on non-alcoholic fatty liver disease through the regulation of SNAI1

**DOI:** 10.1186/s10020-021-00428-7

**Published:** 2022-01-31

**Authors:** Fei Zhao, Jinjing Ke, Wensheng Pan, Hanghai Pan, Miao Shen

**Affiliations:** Health Management Center, Department of Gastroenterology, Zhejiang Provincial People’s Hospital, Affiliated People’s Hospital, Hangzhou Medical College, Hangzhou, 310014 Zhejiang Province China

**Keywords:** Non-alcoholic fatty liver disease, ISL1, KDM6B, SNAI1, Methylation

## Abstract

**Background:**

The increasing incidence of non-alcoholic fatty liver disease (NAFLD) has been reported worldwide, which urges understanding of its pathogenesis and development of more effective therapeutical methods for this chronic disease. In this study, we aimed to investigate the effects of a LIM homeodomain transcription factor, islet1 (ISL1) on NAFLD.

**Methods:**

Male C57BL/6J mice were fed with a diet high in fat content to produce NAFLD models. These models were then treated with overexpressed ISL1 (oe-ISL1), oe-Lysine-specific demethylase 6B (KDM6B), oe-SNAI1, or short hairpin RNA against SNAI1. We assessed triglyceride and cholesterol contents in the plasma and liver tissues and determined the expressions of ISL1, KDM6B and SNAI1 in liver tissues. Moreover, the in vitro model of lipid accumulation was constructed using fatty acids to explore the in vitro effect of ISL1/KDM6B/SNAI1 in NAFLD.

**Results:**

The results showed that the expressions of ISL1, KDM6B, and SNAI1 where decreased, but contents of triglyceride and cholesterol increased in mice exposed to high-fat diet. ISL1 inhibited lipogenesis and promoted lipolysis and exhibited a synergizing effect with KDM6B to upregulate the expression of SNAI1. Moreover, both KDM6B and SNAI1 could inhibit lipogenesis and induce lipolysis. Importantly, the therapeutic effects of ISL1 on in vitro model of lipid accumulations was also confirmed through the modulation of KDM6B and SNAI1.

**Conclusions:**

Taken together, these findings highlighted that ISL1 effectively ameliorated NAFLD by inducing the expressions of KDM6B and SNAI1, which might be a promising drug for the treatment of NAFLD.

**Supplementary Information:**

The online version contains supplementary material available at 10.1186/s10020-021-00428-7.

## Background

Non-alcoholic fatty liver disease (NAFLD) is a dominant chronic liver disease characterized by excess hepatic fat accumulation with increasing prevalence worldwide (Stefan et al. 2019). Excess liver fat is caused by increased delivery of triglycerides (TG) to the liver or a surplus of carbohydrate transformation to TG (Lee et al. 2017). The increased lipid acquisition surpassing lipid disposal, such as, excess fatty acid intake and lipogenesis, leads to steatosis (Ipsen et al. 2018), which subsequently develops to advanced stages with non-alcoholic steatohepatitis (NASH) and/or fibrosis in some cases reporting NAFLD (Pafili and Roden 2020). As previously reported, NAFLD involves a spectrum of lesions that ranges from steatosis to a complicated pattern with hepatocellular damage and inflammatory response (NASH) that is not induced by alcohol intake (Bedossa 2017). A prior study has mentioned the correlation of hepatic steatosis with insulin resistance, diabetes, obesity, and metabolic syndrome, etc. (Willebrords et al. 2015). Despite advancements in understanding the pathophysiology of NAFLD, current available targeted therapies are still insufficient (Singh et al. 2017). Considering the fast-growing global burden of this disease, it is of paramount importance to seek accurate non-invasive biomarkers for diagnosis and prognosis and to develop effective therapeutic targets for advanced NASH, NAFLD or progressive liver disease (Younossi 2019).

Lysine-specific demethylase 6B (KDM6B), also referred to as jumonji domain-containing protein 3 (JMJD3), is a H3K27me3 demethylase that can drive immune responses and evoke cell senescence (Salminen et al. 2014). It has been recently suggested that KDM6B engages in lipid catabolism, thermogenesis, as well as glucose homeostasis (Thibonnier and Esau 2020). Moreover, KDM6B is implicated in metabolic disorder-related diseases, including type 2 diabetes and NASH (Thibonnier et al. 2020; Thibonnier and Esau 2020). Hepatic KDM6B expression is substantially reduced in patients with NAFLD, and it participates in defective autophagy and hepatosteatosis (Byun et al. 2020), demonstrating its promising functional significance in the treatment of NAFLD. Recent studies have focused on the upstream and downstream factors of KDM6B. ISL1 physically interacts with KDM6B to modulate the cardiac progenitor cell differentiation through altering the cardiac epigenome and gene expression (Wang et al. 2016). Additionally, ISL1 has been recognized as a critical factor for the mediation of β cells to control blood sugar and highly expressed ISL1 can improve glucose homeostasis (Liang et al. 2020; Liu et al. 2012). In addition to the available evidence, bioinformatics analysis also revealed aberrant expressions of ISL1 and KDM6B in NAFLD prior to our investigation, and is highly suggestive of their implications in NAFLD. KDM6B can enhance the expression of genes through demethylating H3K27me3 in promoter regions and gene bodies. Specifically, it demethylates H3K27me3 in the SNAI1 promoter region, leading to SNAI1 upregulation (Sui et al. 2019). Hepatocyte-specific ablation of SNAI1 is capable of accelerating insulin-triggered lipogenesis in hepatocytes, aggravating mouse dietary NAFLD, and diminishing insulin resistance (Liu et al. 2018). Given the aforementioned findings, we hypothesized that ISL1 and KDM6b might synergistically control lipid metabolism such as lipogenesis and lipolysis through regulating the expression of SNAI1. We testified this hypothesis in a mouse model of NAFLD induced by a diet high in fat content to identify targets for the prevention and treatment of hepatic steatosis.

## Materials and methods

### Ethics statement

The Animal Ethics Committee of Zhejiang Provincial People’s Hospital, People’s Hospital of Hangzhou Medical College approved all animal experiments to be conducted in this study. We have tried our best to minimize the number of animals used in the tests and reduce their suffering.

### Bioinformatics analysis

The R language “limma” package was used to differentially analyze NAFLD-related microarray dataset GSE89632 in the Gene Expression Omnibus database. Microarray dataset GSE89632 contains 43 samples, including 24 normal samples and 19 NAFLD samples. The expression data from KDM6B was extracted using the boxplot function to draw its expression box plot. MEM was used to analyze the co-expression relationship between KDM6B and other genes.

### Establishment of NAFLD mouse models

Wild-type C57BL/6J mice (male, 22–24 g, 8–10 weeks old) were purchased from HFK BIOSCIENCE (Beijing, China). The mice were reared in a 12-h light–dark cycle under pathogen-free conditions and had free access to food and water. Both ordinary feed and high-fat feed (60 kcal% fat, D12492) were purchased from Research Diets (USA). All the mice were divided into the normal group (mice on standard diet) and the NAFLD group (mice on high-fat diet) (n = 8). Mice were fed for 8 weeks for modeling. At the 8th week, the mice were euthanized, the plasma and liver were obtained to determine the triglyceride (TG) and cholesterol (TC) content, and Oil Red O staining was performed on the liver to evaluate the success of modeling.

To further explore the effect of ISL1/KDM6B/SNAI1 on the degree of steatosis in mice exposed to a high-fat diet, 1 × 10^9^ PFU recombinant adenovirus (AAV8, Hanbio, Shanghai, China) was injected into the tail vein to construct a mouse model harboring gene overexpression/knockdown, 10 weeks after being fed with a high-fat diet. Afterwards, mice were fed with a high-fat diet for another 8 weeks (Zhang et al. 2020), 8 mice in each group (Additional file 1: Fig. S1). Mice were injected with adenovirus harboring overexpressed ISL1 (oe-ISL1), oe-KDM6B, oe-SNAI1, and short hairpin RNA against SNAI1 (sh-SNAI1) as well as their corresponding negative controls (NCs) (oe-NC, sh-NC) (72 mice in total) (Zhang et al. 2020, 2019; Liu et al. 2018). After that, mouse liver tissues were taken for follow-up experiments, including oil red O staining, quantification of triglyceride (TG) and cholesterol (TC) in plasma and liver tissues, hematoxylin–eosin (HE) staining, Immunohistochemistry (IHC), Western blot, and RT-qPCR. Subcutaneous white adipose tissue (WAT) was quantified by measuring the weight of inguinal fat pad (Barrera et al. 2014; Gao et al. 2018).

### Cell culture and treatment

Normal human liver cells THLE-2 were purchased from Ningbo Mingzhou Biotechnology Co., Ltd. (Zhejiang, China; 165861, https://www.mingzhoubio.com/goods-165861.html) and cultured in Dulbecco’s Modified Eagle medium (A4192101; Gibco, USA) containing 10% fetal bovine serum at 37 °C with 5% CO_2_ in an incubator. Human hepatoma cell hepG2 was purchased from the typical culture preservation center of the Chinese Academy of Sciences (Shanghai, China). It was cultured in MEM (41500034; Gibco) in an incubator at 37 °C with 5% CO_2_.

The cells in the logarithmic growth phase were detached and seeded in a 6-well plate. Cell infection was performed on the next day when cell confluence reached 70%. Adenovirus (Shanghai GenePharma Co., Ltd., Shanghai, China) was infected according to the ratio of cells: virus = 1:10. Cells were treated with oe-ISL1, oe-KDM6B, oe-SNAI1, and sh-SNAI1 as well as their corresponding controls (oe-NC, sh-NC).

To construct the in vitro model of lipid accumulation, fatty acids (FA, including 200 μM oleic acid [112-80-1, aladdin, USA] and 100 μM palmitic acid [57-10-3, aladdin]) were added to the cell culture medium to allow cells to be cultured for 72 h. The in vitro models were verified by oil red O staining.

### Oil red O staining

After the mice were euthanized, the liver tissues were removed from the mice’s bodies to observe the extent of fat deposition with the naked eye. Frozen sections were made, and stained with Oil Red O dyeing liquor by following the instructions of the Oil Red O staining kit (O8010, Beijing Solarbio Technology Co., Ltd., Beijing, China). After neutral resin mounting, the accumulation of liver lipid droplets was observed under a microscope. For the in vitro model of lipid accumulation, after the supernatant was discarded, the cells were fixed with 10% formaldehyde for more than 1 h and stained with Oil Red O to observe the lipid accumulation in the cells.

### Measurement of TG and TC contents

After the mice were euthanized, the mouse plasma was taken for TG and TC content quantification by following the operation manual of the TG quantification kit (A110-1–1, Nanjing Jiancheng Bioengineering Institute, Nanjing, Jiangsu, China) and the TC quantification kit (A111-1-1, Nanjing Jiancheng Bioengineering Institute), followed by the determination of the absorbance value at 510 nm. The levels of serum alanine aminotransferase (ALT) and aspartate aminotransferase (AST) were measured by automatic clinical analyzer (Hitachi 7180, Tokyo, Japan). The mice’s livers were chopped, suspended in phosphate buffer saline (PBS) solution, and lysed with ultrasound until the cells were completely disintegrated. The suspension was taken for quantification as described above, and bicinchoninic acid (BCA) was used to quantify protein content (Susutlertpanya et al. 2019).

### HE staining

The tumor tissues of nude mice were fixed, paraffin embedded, sectioned at 4 μm, dewaxed with xylene, and rehydrate with ethanol [xylene (I) for 5 min, xylene (II) for 5 min, 100% ethanol for 2 min, 95% ethanol for 1 min, 80% ethanol for 1 min, 75% ethanol for 1 min, and distilled water for 2 min]. Next, the sections were stained with hematoxylin for 5 min, differentiated for 30 s, immersed in tap water for 15 min, then put in eosin solution for 2 min, conventionally dehydrated [95% ethanol (I) for 1 min, 95% ethanol (II) for 1 min, 100% ethanol (I) for 1 min, 100% ethanol (II) for 1 min, xylene carbonate for 1 min, xylene (I) for 1 min, and xylene (II) for 1 min], cleared, and sealed. Finally, the sections were observed under an inverted microscope (XSP-8CA, Shanghai Optical Instrument Factory, Shanghai, China).

### IHC

Paraffin sections were deparaffinized to water, dehydrated with alcohol gradient, washed in tap water for 2 min, treated with H_2_O_2_ containing 3% methanol for 20 min and subjected to antigen retrieval. Afterwards, the sections were blocked with normal goat serum blocking solution (Shanghai Haoran Biotechnology Co., Ltd, Shanghai, China) for 20 min. Next, the sections were incubated with primary antibodies against ISL1 (ab178400, 1: 200, Abcam, Cambridge, UK) overnight at 4 °C. The sections were later incubated with goat anti-rabbit secondary antibody immunoglobulin G (IgG; ab6785, 1: 1000, Abcam) for 20 min at 37 °C and with horseradish peroxidase (HRP)-labeled streptavidin protein working solution (0343-10000U, Imunbio Co., Ltd., Beijing, China) at 37 °C for 20 min. The sections were then developed by 3,3’-diaminobenzidine (DAB) (ST033, Guangzhou WHIGA Technology Co., Ltd., Guangzhou, China), counter-stained with hematoxylin (PT001, Shanghai Bogoo Biotechnology Co., Ltd., Shanghai, China) for 1 min, reverted to blue color by 1% ammonia water, dehydrated by gradient alcohol, cleared by xylene, sealed by neutral resin and observed under a microscope with 5 randomly selected high-powered fields.

### Western blot analysis

Radio immunoprecipitation assay lysis, containing protease inhibitor (BOSTER Biological Technology Co., Ltd., Wuhan, Hubei, China), was added to lyse tissues and cells to extract the total protein content of the samples. The total protein concentration of each sample was determined using a BCA kit (BOSTER). The protein was boiled at 95 °C for 10 min, separated by sodium dodecyl sulfate (SDS)-polyacrylamide gel electrophoresis and transferred onto a polyvinylidene fluoride membrane. The membrane was left to block with 5% bovine serum albumin for 2 h and incubated with diluted primary antibodies overnight at 4 °C. Following washing, the membrane was incubated with HRP-labeled goat anti-rabbit secondary antibodies (ab205719, 1: 2000, Abcam) for 1 h at room temperature. After washes, the membrane was immersed in enhanced chemiluminescence solution (EMD Millipore, USA) for 1 min at room temperature. The membrane was covered with plastic wrap, developed, and imaged after the chemiluminescence solution was removed. β-actin was used as an internal reference, and the gray values of the bands were analyzed by Western blot analysis. The primary antibodies used in the study were: anti-stearoyl-CoA desaturase 1 (SCD1) (#2794; 1:1000; Cell Signaling Technology, USA), anti-FAS (#3189; 1:1000; Cell Signaling Technology), anti-Acetyl-CoA carboxylase 1 (ACC1) (#4190; 1:1000; Cell Signaling Technology), anti-Sterol-regulatory element binding protein 1 (SREBP1) (ab3259; 1:1000; Abcam), anti-Adipose triglyceride lipase (ATGL) (#2439; 1:1000; Cell Signaling Technology), anti-Monoacylglycerol lipase (MGL) (ab124796; 1:1000; Abcam), anti-hormone-sensitive lipase (HSL) (NB110-37,253; 1:1000; Novus, USA), anti-β-actin (#4970; 1:1000; Cell Signaling Technology), anti-ISL1 (ab178400; 1:1000; Abcam), anti-KDM6B (#3457; 1:1000; Cell Signaling Technology), anti-SNAI1 (#3879; 1:1000; Cell Signaling Technology), anti-c-caspase 3 (ab184787, 1:1000; Abcam), and anti-c-caspase 7 (ab255818; 1:1000; Abcam).

### Reverse transcription quantitative polymerase chain reaction (RT-qPCR)

The total RNA content was extracted from cells and tissue samples with Trizol reagent (Invitrogen), and was reversely transcribed into complementary DNA (cDNA) by following the protocols of thePrime Script™ RT (Takara Biotechnology, China). RT-qPCR experiments were performed using TransStart Tip Green qPCR SuperMix (TransGen Biotech, Beijing, China). β-actin was used as an internal reference, and the primer sequences are shown in Additional file 8: Table S1. Fold alterations in expression were analyzed with the 2^−∆∆Ct^ method.

### Co-immunoprecipitation (Co-IP)

Cells were lysed in the lysis buffer containing 1 mL protease inhibitor mixture (BOSTER), allowed to sit on ice for 15 min, centrifuged at 12,000 rpm for 10 min, and collected. Next, the cell lysate was left to mix with anti-ISL1/anti-KDM6B and 25 μL protein A sepharose at 4 °C overnight. Afterwards, the beads were washed three times with the lysis buffer, added with 2 × SDS loading buffer, heated at 95 °C for 10 min, and centrifuged. Western blot analysis was used to analyze KDM6B/ISL1 levels. Before Co-IP, 10% supernatant was collected for Input detection.

### Immunofluorescence

Immunofluorescence kit (Beyotime Biotechnology, Shanghai, China) was used for immunofluorescence signal detection. anti-ISL1 antibodies (ab178400, Abcam) and anti-KDM6B antibodies (#3457; 1:1000; Cell Signaling Technology) were applied to detect the corresponding protein. The results were observed under a confocal microscope (Leica, Wetzlar, Germany) (ISL1 protein: red spots, KDM6B protein: green spots) with 4ʹ,6-Diamidino-2-phenylindole used to label nuclear DNA (blue).

### Chromatin immunoprecipitation assay (ChIP)

Cells were fixed with formaldehyde for 10 min to produce DNA–protein cross-links. Cells were sonicated 15 times for 10 s each to produce DNA fragments. The sheared chromatin lysate was incubated with IgG (ab6785, 1:1000, Abcam), anti-KDM6B (#3457; 1:1000; Cell Signaling Technology), anti-H3K27me3 (#9733; 1:1000; Cell Signaling Technology), anti-H3K9me3 (#13969; 1:1000; Cell Signaling Technology), and anti-H3K36me3 (#4909; 1:1000; Cell Signaling Technology) overnight at 4 °C. DNA–protein complex was precipitated by Protein Agarose/Sepharose, and centrifuged at 12,000×*g* for 5 min, followed by washing. Subsequently, reverse cross-linking was performed at 65 °C. Finally, the samples were subjected to DNA purification with phenol/chloroform and DNA fragment recycle. The binding of KDM6B with SNAI1 promoter was detected by qPCR based on specific primers of the SNAI1 promoter region (Forward: 5ʹ-GGCACGGCCTAGCGAGT-3ʹ; Reverse: 5ʹ-AGTGGTCGAGGCACTGGG-3ʹ).

### Statistical analysis

All data in this study were analyzed using the SPSS 21.0 (IBM, Armonk, NY, USA) software. Measurement data were displayed as the mean ± standard deviation. An unpaired *t*-test was used for data comparison between two groups, one-way analysis of variance (ANOVA) was used for data comparison among multiple groups followed by Tukey’s post-hoc test. A value of* p* < 0.05 was considered statistically significant. The correlation between two genes was determined by Pearson correlation analysis.

## Results

### SNAI1 and ISL1 have a significant co-expression relationship with KDM6B in NAFLD samples

**Fig. 1:**
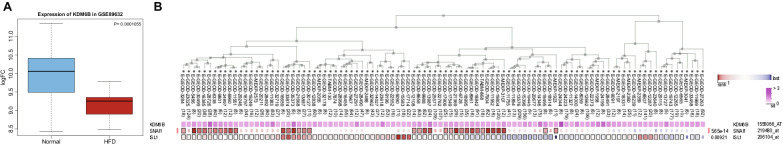
Bioinformatics analysis of the co-expression relationship among ISL1/KDM6B/SNAI1 in NAFLD samples. **A** The box plot of the expression data of KDM6B in the GSE89632 dataset, the blue box on the left represents the expression of normal samples, and the red box on the right represents the expression of NAFLD. **B** MEM predicted significant co-expression of KDM6B with ISL1 and SNAI1

Existing studies have shown that KDM6B is poorly expressed in NAFLD (Byun et al. 2020). By performing differential analysis of NAFLD-related microarray dataset GSE89632, we confirmed that KDM6B was poorly expressed in NAFLD (Fig. 1A). A previous study had reported that ISL1 could cooperate with KDM6B to mediate downstream genes, KDM6B could remove the methylation of SNAI1, and the low expression of SNAI1 promoted fat synthesis of NAFLD (Liu et al. 2018; Sui et al. 2019; Wang et al. 2016). A significant co-expression of SNAI1 and ISL1 with KDM6B was determined through co-expression analysis using MEM platform (Fig. 1B). Therefore, we speculated that ISL1 may mediate SNAI1 methylation by cooperating with KDM6B to affect the occurrence of NAFLD.

### Poorly expressed ISL1 in NAFLD

**Fig. 2:**
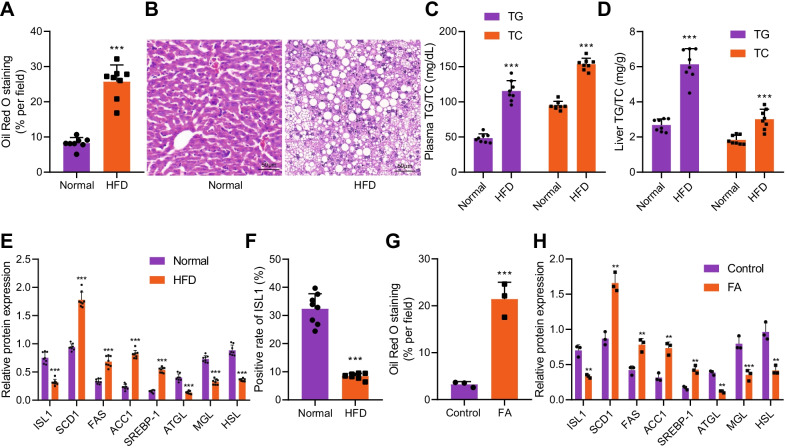
Decreased ISL1 was observed in NAFLD. **A** Oil red O staining was applied to detect liver lipid accumulation in mice exposed to high-fat diet. **B** Morphology of liver tissues detected by HE staining. **C** Detection of plasma contents of TG and TC in mice exposed to high-fat diet. **D** Detection of contents of TG and TC in liver tissues of mice exposed to high-fat diet. **E** Western blot analysis was conducted to detect the expression of ISL1, lipid synthesis- and lipolysis-related genes in liver tissue of mice exposed to high-fat diet. **F** Immunohistochemistry was used to detect the expression of ISL1 in liver tissues of mice exposed to high-fat diet. **G** Oil red O staining was performed to detect lipid accumulation. **H** Western blot analysis was used to detect the expressions of ISL1, lipid synthesis- and lipolysis-related genes in an in vitro model of lipid accumulation. **p < 0.01 vs. Control (cells without treatment); ***p < 0.001 vs. Normal (mice on standard diet) or Control (cells without treatment). n = 8 mice in each group

In order to clarify the expression and role of ISL1 in NAFLD, we constructed a mouse model of NAFLD by giving mice a diet high in fat content. From the fourth week to the eighth week of modeling, the weight of mice on a high-fat diet was higher than that of mice on the standard diet (Additional file 2: Fig. S2A). There was no significant difference in food intake between NAFLD modeled mice and normal mice (Additional file 2: Fig. S2B), and the subcutaneous fat weight of NAFLD modeled mice was significantly higher than that of normal mice (Additional file 2: Fig. S2C). Oil red O staining revealed that mice with high-fat diet showed increased accumulation of lipid droplets in liver tissue compared with that of the standard diet (Fig. 2A; Additional file 3: Fig. S3A). HE staining showed enlarged liver cells in NAFLD accompanied by diffuse lipid vacuolation (Fig. 2B), suggesting the successful establishment of NAFLD mouse models. In addition, elevated contents of TG and TC in liver tissues of NAFLD also demonstrated the successful establishment of NAFLD mouse models (Fig. 2C, D). Though Western blot analysis and IHC, we found that mice with high-fat diet presented with increased expressions of lipid synthesis-related genes (SREBP-1, ACC1, FAS, and SCD1), decreased expressions of lipolysis-related genes (ATGL, MGL, and HSL), and down-regulated expression of ISL1 (Fig. 2E, F; Additional file 3: Fig. S3B, Additional file 4: Fig. S4A). Moreover, we used FA to treat liver cell line THLE-2 to establish an in vitro model of lipid accumulation (Fig. 2G; Additional file 3: Fig. S3C), followed by the detection of the expression of lipid synthesis-related genes, lipolysis-related genes, and ISL1 in the cells. The obtained results were consistent with those found in mouse models (Fig. 2H; Additional file 4: Fig. S4B). In conclusion, ISL1 was poorly expressed in NAFLD.

### ISL1 inhibits lipogenesis and promotes lipolysis

**Fig. 3:**
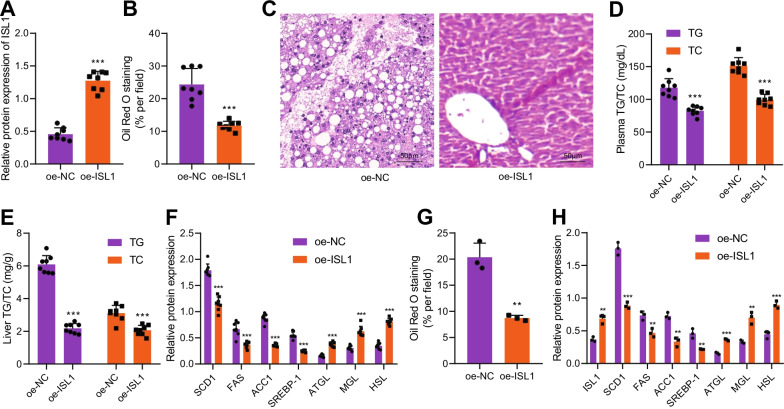
ISL1 led to inhibited lipogenesis and induced lipolysis. **A** Western blot analysis was used to detect the ISL1 overexpression efficiency in liver tissues of mice exposed to high-fat diet. **B** Oil red O staining was applied to detect the effect of overexpressed ISL1 on liver lipid accumulation in mouse model. **C** Morphology of liver tissues detected by HE staining. **D** Effect of overexpressing ISL1 on plasma contents of TG and TC in mice exposed to high-fat diet. **E** Effect of overexpressing ISL1 on the contents of TG and TC in liver tissues of mice exposed to high-fat diet. **F** Western blot analysis was conducted to detect the effect of ISL1 on lipid synthesis- and lipolysis-related genes in liver tissues of mice exposed to high-fat diet. **G** Oil red O staining was performed to detect the effect of ISL1 on lipid accumulation. **H** Western blot analysis was used to detect the effect of ISL1 on the expressions of lipid synthesis- and lipolysis-related genes in an in vitro model of lipid accumulation. **p < 0.01, ***p < 0.001 vs. oe-NC treatment. n = 8 mice in each group

In the following experiments, we investigated the role of ISL1 in the development of NAFLD. NAFLD mouse models were injected with oe-ISL1 adenovirus into the mice’s tail veins, and mice livers were extracted to verify the overexpression of ISL1 (Fig. 3A, Additional file 4: Fig. S4C). We found that after overexpressing ISL1, the weight of the mice decreased (Additional file 2: Fig. S2D), food intake showed no significant difference (Additional file 2: Fig. S2B), and the subcutaneous fat weight was reduced (Additional file 2: Fig. S2C). Results from Oil red O and HE staining showed that overexpressing ISL1 reduced the degree of liver steatosis, reduced the volume of liver cells in liver tissues with fewer lipid vacuolation accompanied by decreased TG and TC contents (Fig. 3B–E; Additional file 3: Fig. S3D). Western blot analysis demonstrated that after overexpressing ISL1 decreased the expression of lipid synthesis-related genes in the liver tissues of high-fat mice models, while increasing the expression of lipolysis-related genes, which was consistent with the above experimental results (Fig. 3F; Additional file 4: Fig. S4D).

Furthermore, in the in vitro model of lipid accumulation, treatment of adenovirus expressing overexpressed ISL1 led to a reduced content of lipid droplets in cells (Fig. 3G; Additional file 3: Fig. S3E), accompanied by decreased expression of lipid synthesis-related genes and increased expression of lipolysis-related genes (Fig. 3H; Additional file 4: Fig. S4E). The above experimental results showed that ISL1 inhibited lipogenesis and promoted lipolysis.

### ISL1 cooperates with KDM6B to upregulate SNAI1

**Fig. 4:**
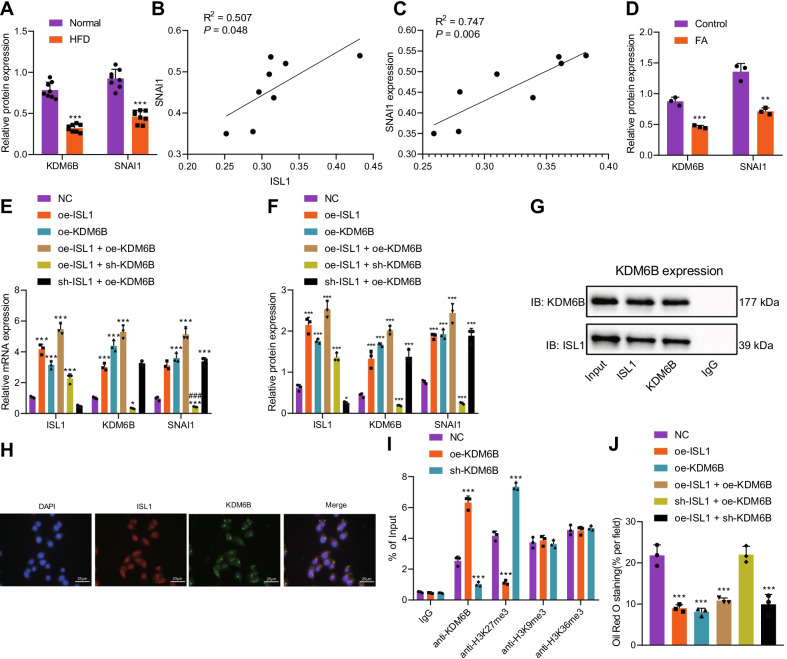
Synergistic effects of ISL1 and KDM6B on SNAI1. **A** Expressions of KDM6B and SNAI1 in liver tissues of mice exposed to high-fat diet detected by Western blot analysis. **B** The correlation between ISL1 and SNAI1 in mouse liver tissues was analyzed by Pearson correlation analysis. **C** The correlation between KDM6B and SNAI1 in mouse liver tissues was analyzed by Pearson correlation analysis. **D** Expressions of KDM6B and SNAI1 in an in vitro model of lipid accumulation determined by Western blot analysis. **E** RT-qPCR was used to detect the expression of ISL1/KDM6B/SNAI1 in THLE-2 cells after overexpressing or knockdown of ISL1/KDM6B. **F** Western blot was used to detect the expression of ISL1/KDM6B/SNAI1 in THLE-2 cells after overexpressing/knockdown of ISL1/KDM6B. **G** Co-IP detection of the binding between ISL1 and KDM6B in vitro. **H** Immunofluorescence detection of the localization of ISL1 and KDM6B in cells. **I** Oil red O staining shows steatosis in response to artificial modulation of ISL1 and KDM6B expression. **J** ChIP detection of effect of overexpressing/knockdown of KDM6B on KDM6B enrichment and methylation degree in SNAI1 promoter region. *p < 0.05, ***p < 0.001 vs. NC treatment; ^###^*p* < 0.001 vs. oe-ISL1 treatment. Cell experiment was repeated 3 times independently. n = 8 mice in each group

A previous study found that ISL1 and the epigenetic modification enzyme, KDM6B, have a synergistic effect, both of which can regulate and promote their own expressions; both also synergistically mediate the expressions of downstream genes (Wang et al. 2016). KDM6B can upregulate SNAI1 by catalyzing the demethylation of the promoter region of the lipid metabolism-related gene SNAI1, therefore exerting corresponding biological functions (Sui et al. 2019). However, the relationship between ISL1 and KDM6B/SNAI1 and the role of KDM6B/SNAI1 in NAFLD have not been reported. We firstly determined the expressions of KDM6B and SNAI1 in the liver tissues of mice exposed to a high-fat diet, and the results showed that both were poorly expressed (Fig. 4A; Additional file 4: Fig. S4F), which was consistent with the low expression of ISL1 as verified above. Additionally, a significant positive correlation was observed between KDM6B/ISL1 and SNAI1 (Fig. 4B, C), suggesting that the ISL1/KDM6B/SNAI1 axis may play an important role in the development of NAFLD.

Next, we tried to explore the regulatory relationship between ISL1 and KDM6B/SNAI1 in liver tissues. THLE-2 cells were treated with FA to develop an in vitro model of lipid accumulation, followed by quantification of KDM6B/SNAI1, results of which revealed downregulated levels of KDM6B/SNAI1 in the presence of FA (Fig. 4D; Additional file 4: Fig. S4G). We then overexpressed ISL1 and KDM6B simultaneously or separately, and the results showed that overexpressing ISL1 or KDM6B alone had increased the expression of SNAI1, confirming the existing interacting relationship between ISL1 and KDM6B. Additionally, the high expressions of ISL1 and KDM6B elevated the expression of ISL1/KDM6B/SNAI1, which also confirmed the mutual promoting relationship between ISL1 and KDM6B (Fig. 4E, F; Additional file 4: Fig. S4H). Co-IP and immunofluorescence assays also demonstrated the binding interaction between ISL1 and KDM6B in cells (Fig. 4G–H), confirming the interaction and mutual regulation of the two. Knocking down KDM6B while overexpressing ISL1 inhibited SNAI1 expression; knocking down ISL1 while overexpressing KDM6B would not inhibit the expression of SNAI1, indicating that the upstream regulator that directly interacted with SNAI1 was KDM6B instead of ISL1 (Fig. 4E, F). Oil red O staining showed that knocking down KDM6B while overexpressing ISL1 inhibited steatosis, while knocking down ISL1 while overexpressing KDM6B did not inhibit steatosis (Fig. 4I; Additional file 3: Fig. S3F).

Since KDM6B played a regulatory role by mainly mediating the demethylation of target genes, we further tested the effect of overexpression/knockdown of KDM6B on the KDM6B enrichment and methylation degree in the promoter region of SNAI1 through ChIP, and the results revealed that after overexpressing KDM6B, the KDM6B enrichment in the SNAI1 promoter region was significantly increased, and the methylation degree was reduced; knocking down KDM6B led to the opposite effect (Fig. 4J). These results suggested that KDM6B enhanced SNAI1 expression by augmenting SNAI1 promoter demethylation. In addition, we also found that only H3K27me3 modification changed with overexpression/knockdown of KDM6B, while H3K9me3 and H3K36me3 were basically unchanged, suggesting that KDM6B had mainly affected the H3K27me3 modification of SNAI1. The aforementioned results indicated the presence of an existing synergistic effect of ISL1 and KDM6B, and that KDM6B enhancing SNAI1 expression by regulating SNAI1 promotor demethylation.

### KDM6B and SNAI1 inhibit lipogenesis and induce lipolysis

**Fig. 5:**
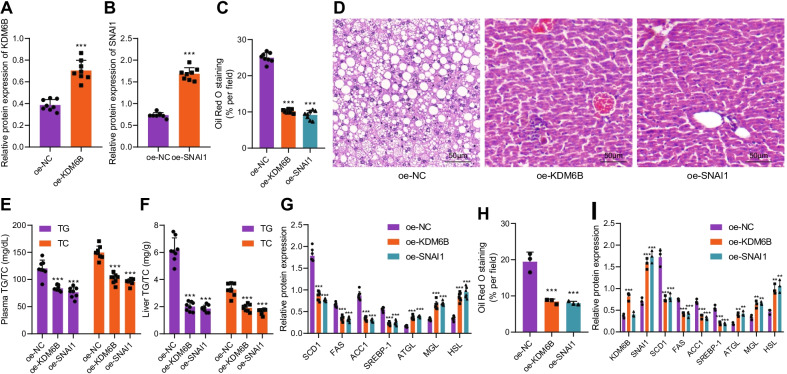
KDM6B and SNAI1 repressed lipogenesis and promoted lipolysis. **A** Western blot analysis was used to detect the KDM6B overexpression efficiency in liver tissues of mice exposed to high-fat diet. **B** Western blot analysis was used to detect the SNAI1 overexpression efficiency in liver tissues of mice exposed to high-fat diet. **C** Oil red O staining was applied to detect the effect of overexpressing KDM6B and SNAI1 on liver lipid accumulation in mouse model. **D** Morphology of liver tissues affected by overexpressing KDM6B and SNAI1 detected by HE staining. **E** Effect of overexpressing KDM6B and SNAI1 on plasma contents of TG and TC in mice exposed to high-fat diet. **F** Effect of overexpressing KDM6B and SNAI1 on the contents of TG and TC in liver tissues of mice exposed to high-fat diet. **G** Western blot analysis was conducted to detect the effect of KDM6B and SNAI1 on lipid synthesis- and lipolysis-related genes in liver tissues of mice exposed to high-fat diet. **H** Oil red O staining was performed to detect the effect of KDM6B and SNAI1 on lipid accumulation. **I** Western blot analysis was used to detect the effect of overexpressed KDM6B and SNAI1 on the expressions of KDM6B, SNAI1, lipid synthesis- and lipolysis-related genes in an in vitro model of lipid accumulation. ***p < 0.001 vs. oe-NC treatment. Cell experiment was repeated 3 times independently. n = 8 mice in each group

We then investigated the effect of KDM6B and SNAI1 on the development of NAFLD. Overexpression efficiency of oe-KDM6B and oe-SNAI1 treatment was first confirmed by Western blot analysis (Fig. 5A, B; Additional file 4: Fig. S4I, J). We found that simultaneous overexpressing KDM6B and SNAI1 had reduced the weight of mice (Additional file 2: Fig. S2E), whereas food intake showed no significant difference (Additional file 2: Fig. S2B), and the subcutaneous fat weight was reduced (Additional file 2: Fig. S2C), alleviated liver steatosis by reducing lipid droplet accumulation (Fig. 5C; Additional file 3: Fig. S3G). Further, simultaneous overexpressing KDM6B and SNAI1 also decreased the volume of liver cells in liver tissues and lipid vacuolation (Fig. 5D) and TG and TC contents (Fig. 5E, F). Additionally, overexpressing KDM6B/SNAI1 decreased the expression of lipid synthesis-related genes and increased the expression of lipolysis-related genes (Fig. 5G; Additional file 4: Fig. S4K). These results suggested that the overexpression of KDM6B/SNAI1 ameliorated NAFLD.

Moreover, similar conclusions were also obtained in the in vitro model of lipid accumulation by overexpressing KDM6B and SNAI1 (Fig. 5H, I; Additional file 3: Fig. S3H, Additional file 4: Fig. S4L). The above experimental results clarified that KDM6B and SNAI1 inhibited lipogenesis and promoted lipolysis, and overexpressing KDM6B or SNAI1 protected against liver steatosis.

### ISL1 cooperates with KDM6B to relieve NAFLD by upregulating SNAI1

**Fig. 6:**
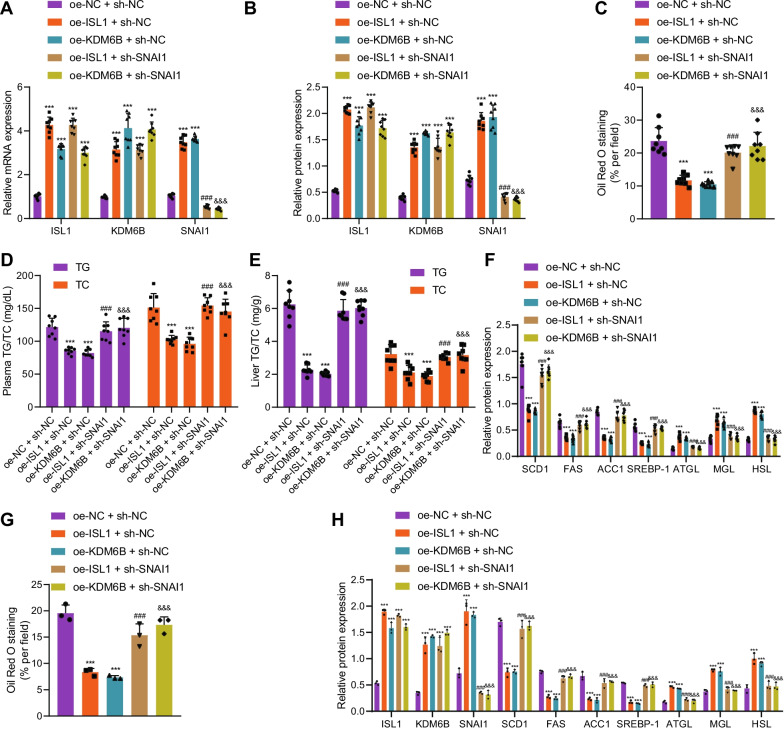
Synergistic alleviative effects of ISL1 and KDM6B on NAFLD by upregulating the expression of SNAI1. **A** RT-qPCR was used to detect the effect of overexpressing ISL1/KDM6B and knockdown of SNAI1 on the expression levels of ISL1, KDM6B, and SNAI1 in the liver tissues of mice exposed to high-fat diet. **B** Western blot analysis was applied to detect the effect of overexpressing ISL1/KDM6B and knockdown of SNAI1 on the expression levels of ISL1, KDM6B, and SNAI1 in the liver tissues of mice exposed to high-fat diet. **C** Oil red O staining was applied to detect the effect of overexpressing ISL1/KDM6B and knockdown of SNAI1 on liver lipid accumulation in mouse model. **D** Effect of overexpressing ISL1/KDM6B and knockdown of SNAI1 on the plasma contents of TG and TC in mice exposed to high-fat diet. **E** Effect of overexpressing ISL1/KDM6B and knockdown of SNAI1 on the contents of TG and TC in liver tissues of mice exposed to high-fat diet. **F** Western blot analysis was conducted to detect the effect of overexpressing ISL1/KDM6B and knockdown of SNAI1 on lipid synthesis- and lipolysis-related genes in liver tissues of mice exposed to high-fat diet. **G** Oil red O staining was performed to detect the effect of overexpressing ISL1/KDM6B and knockdown of SNAI1 on lipid accumulation. **H** Western blot analysis was used to detect the effect of overexpressing ISL1/KDM6B and knockdown of SNAI1 on the expressions of ISL1, KDM6B, SNAI1, lipid synthesis- and lipolysis-related genes in the in vitro model of lipid accumulation. ***p < 0.001 vs. oe-NC + sh-NC treatment; ^###^*p* < 0.001 vs. oe-ISL1 + sh-NC treatment; ^&&&^*p* < 0.001 vs. oe-KDM6B + sh-NC treatment. Cell experiment was repeated 3 times independently. n = 8 mice in each group

The aforementioned experimental results have determined the relieving effects of ISL1/KDM6B/SNAI1 on NAFLD and the regulatory mechanism among the three. The focus of the investigation was then shifted to the involvement of SNAI1 in the functional role of the ISL1/KDM6B axis. In the hepG2 cell model, the expression of ISL1/KDM6B/SNAI1 was downregulated, and overexpression of ISL1/KDM6B/SNAI1 could attenuate lipid droplet accumulation (Additional file 5: Fig. S5A, B). We found that overexpressing ISL1 or KDM6B had increased the expressions of ISL1 and KDM6B, again verifying the mutual regulation between the two; simultaneous treatment of overexpressed ISL1/KDM6B and knocked down SNAI1 had no effect on the expression of the first two, and the expression of SNAI1 was decreased significantly, which also confirmed the upstream and downstream regulatory relationship between the three (Fig. 6A, B; Additional file 4: Fig. S4M). Our experimental results also demonstrated that overexpressing ISL1 or KDM6B significantly reduced the weight of mice (Additional file 2: Fig. S2F), the degree of liver steatosis (Fig. 6C; Additional file 3: Fig. S3I), as well as the contents of TG and TC (Fig. 6D, E), and after overexpression of ISL1 and inhibition of SNAI1, or overexpression of KDM6B and inhibition of SNAI1, food intake showed no significant difference (Additional file 2: Fig. S2B), and the subcutaneous fat weight was enhanced (Additional file 2: Fig. S2C). While knocking down SNAI1 at the same time nullified this therapeutic effect. Results from the Western blot analysis revealed that overexpressing ISL1 or KDM6B reduced the expression of lipid synthesis-related genes and increased the expression of lipolysis-related genes, while knocking down SNAI1 nullified this effect (Fig. 6F; Additional file 4: Fig. S4N). In addition, similar results were obtained in the in vitro model of lipid accumulation (Fig. 6G, H; Additional file 3: Fig. S3J, Additional file 4: Fig. S4O). To explore the mechanism of ISL1/KDM6B/SNAI1 pathway to ameliorate NAFLD, we also detected the key proteins in lipid oxidation, autophagy related pathway. After overexpression of ISL1/KDM6B/SNAI1, the expression of CPT1A, CPT2, and ACADM was upregulated, expression of LC3, ATG5 and ATG6 was upregulated, while p62 expression was downregulated (Additional file 6: Fig. S6).

The results showed that AST and ALT levels in NAFLD modeled mice were significantly higher than those in normal mice. After overexpression of ISL1/KDM6B/SNAI1, AST and ALT levels decreased, while overexpressing ISL1 simultaneously inhibiting SNAI1, or overexpressing KDM6B simultaneously inhibiting SNAI1, AST and ALT levels increased (Additional file 7: Fig. S7A, B). Western blot showed that c-caspase 3 and c-caspase 7 levels in NAFLD modeled mice were higher than those in normal mice. After overexpression of ISL1/KDM6B/SNAI1 in NAFLD mice, c-caspase 3 and c-caspase 7 levels were decreased, but c-caspase 3 and c-caspase 7 levels were increased when overexpressing ISL1 simultaneously inhibiting SNAI1, or overexpressing KDM6B simultaneously inhibiting SNAI1 (Additional file 7: Fig. S7C).

The above results concluded that ISL1 cooperated with KDM6B to ameliorate NAFLD by upregulating SNAI1.

## Discussion

**Fig. 7:**
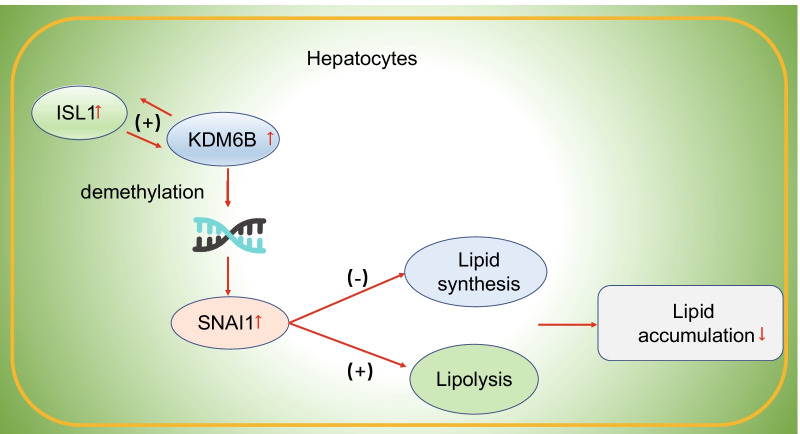
Schematic diagram concerning the role of ISL1 in NAFLD. ISL1 cooperated with KDM6B to improve NAFLD through reduction of the methylation level of SNAI1

NAFLD has emerged as a primary challenge due to its high prevalence, difficult diagnosis, intricate pathogenesis, and the lack of recognized treatment strategies (Neuschwander-Tetri 2017). Recently, a series of therapeutics are directed at pathogenic processes including inflammatory response, hepatic lipid accumulation, injury and fibrogenesis (Ipsen et al. 2018; Rotman and Sanyal 2017). Hence, identification of mechanisms underlying these processes is potentially conducive to the development of target approaches. It has been addressed in the last decade that histone demethylases are crucial regulators of hepatic lipogenesis as well as lipid influx and metabolism (Manuel and Haeusler 2020; Nagaoka et al. 2015). In this study, we identified that ISL1 had interacted with H3K27me3 demethylase KDM6B to promote the expression of SNAI1, by which lipogenesis and steatosis were attenuated while lipolysis was promoted in the mice models of NAFLD (Fig. 7).

First of all, our study demonstrated that ISL1 was poorly expressed in mice models with NAFLD. We also established a model of lipid accumulation using FA induction to mimic the pathogenic conditions in vitro. At the cellular level, re-expression of ISL1 contributed to the suppression of lipogenesis and promotion of lipolysis, as demonstrated by the reductions in lipogenic gene levels (SREBP-1, ACC1, FAS and SCD1) and elevations in lipolytic genes levels (ATGL, MGL, and HSL). Although there is no direct evidence regarding the relationship between ISL1 and hepatic lipogenesis in previous studies, ISL1 has been reported to correlate with lipid droplet formation in cardiac stem cells (Zhang et al. 2018). Furthermore, deletion of ISL1 in the intestine causes reduced expression levels of incretin hormones glucagon-like peptide-1 and glucose-dependent insulinotropic polypeptide, thus contributing to lipid malabsorption and impaired glucose tolerance (Terry et al. 2014). HSL has been used to evaluate lipolysis by hydrolyzing diacylglycerol into monoacylglycerol during TG breakdown (Jha et al. 2014). Elevated levels of HSL and ATGL are indicative of promoted lipolysis and reduced lipogenesis induced by erythropoietin and darbepoetin alpha for alleviation of NAFLD (Tsuma et al. 2019). Also, sulforaphane has been demonstrated to activate lipolysis by upregulating ATGL and HSL (Lei et al. 2019). In addition to HSL, MGL is known to catalyze lipolysis centered in adipose tissues as well (Quiroga and Lehner 2018). To validate the effect of ISL1, we used a murine model and the results confirmed that overexpressing ISL1 attenuated lipogenesis and steatosis after NAFLD models were established.

Additionally, a previous study suggested the presence of the co-expression of ISL1 and KDM6B in NAFLD and that ISL1 could interact with KDM6B (Wang et al. 2016). KDM6B is known as one of the genes participating in lipid oxidation, thermogenesis or glucose homeostasis (Thibonnier and Esau 2020). It is also reported that KDM6B is involved in controlling the pro-fibrotic transcriptional profile of peritoneal foam cells and its deletion blocks the activation of pro-fibrotic pathways (Neele et al. 2017). Liver-specific deficiency of KDM6B leads to intrinsic defects in β-oxidation, resulting in hepatosteatosis, glucose and insulin intolerance, whereas its overexpression selectively inhibits lipid levels without increasing glucose levels in patients with obesity, hepatosteatosis, and type 2 diabetes (Seok et al. 2018). In the presence of FGF21, KDM6B can epigenetically activate autophagic markers, such as Tfeb, Atg7, Atgl, and Fgf21 through demethylation, contributing to autophagy-mediated lipid degradation (Neele et al. 2017). Hence, we speculated that ISL1 could interact with KDM6B to reduce lipid synthesis and promote degradation by inducing anti-lipogenic pathways.

Our data provided evidence that KDM6B could be enriched in the promoter region of SNAI1, thus upregulating the expression of SNAI1 via demethylation. SNAI1 can repress the activity of fatty acid synthase promoter through recruitment of HDAC1/2 and epigenetically downregulates lipogenic genes (Liu et al. 2018). SNAI1 also represses adipogenesis by downregulating Nr2f6, which in turn enhances the expression of anti-adipogenic interleukin-17 (Pelaez-Garcia et al. 2015). As a mediator of transforming growth factor β1-induced epithelial-to-mesenchymal transition, SNAI1 inhibits lipogenic regulators including fatty acid synthase and carbohydrate-responsive-element-binding protein (Jiang et al. 2015). In addition, SNAI1 can repress the level of adiponectin seven days after adipogenesis and reduce levels of peroxisome proliferators-activated receptor γ and CCAAT/enhancer binding protein α that control adipogenesis (Park et al. 2012). Both the in vitro and in vivo results suggested that KDM6B could alleviate lipogenesis and steatosis by upregulating SNAI1. More importantly, some patients with NAFLD have been reported to suffer from NASH, characterized by hepatitis and hepatic injury, further leading to fibrosis or cirrhosis and eventually developing hepatocellular carcinoma (Sumida and Yoneda 2018; Kolodziejczyk et al. 2019). However, the implication of the ISL1/KDM6B/SNAI1 axis in NAFLD remains under-studied. The experimental data in our study revealed that the ISL1/KDM6B/SNAI1 axis restrict progression of NAFLD, providing theoretical basis on the pathogenesis of NAFLD and it being a potential target for treating NAFLD. Hence, the diagnostic and therapeutic implication of the ISL1/KDM6B/SNAI1 axis in NAFLD warrants further investigations.

Hepatic steatosis is the result that lipid acquisition exceeds lipid treatment, that is, fatty acid uptake and new fat production exceed fatty acid oxidation and output. Hepatic steatosis leads to systemic metabolic disorder and adverse effects on multiple organs (Ipsen et al. 2018). NAFLD ranges from simple steatosis and NASH with varying degrees of fibrosis to liver cirrhosis (liver cirrhosis is the main risk factor for hepatocellular carcinoma) (Mato et al. 2019). NAFLD is associated with obesity, type 2 diabetes and dyslipidemia (Jensen et al. 2018; Friedman et al. 2018). In this study, we constructed NAFLD mouse model, overexpression/knockdown specific gene mouse model by tail vein injection of adeno-associated virus, and NAFLD cell model by adding fatty acids to human normal hepatocyte THLE-2 medium. We verified that transcription factor ISL1 cooperated with demethylase KDM6B to upregulate lipid metabolizing enzyme SNAI1, so as to protect against improve NAFLD, including reducing the degree of hepatic steatosis, plasma lipid concentration, and lipid accumulation in cells. We also found that ISL1/KDM6B/SNAI1 pathway played a role in regulating body weight and subcutaneous fat accumulation in mice. Considering that NAFLD is closely related to metabolism and is affected by systemic and adipose tissue metabolic imbalance, whether ISL1/KDM6B/SNAI1 pathway directly or indirectly affects NAFLD remains to be studied.

Taken together, this study suggests the anti-lipogenic effect of ISL1 and KDM6B in NAFLD, which is achieved by KDM6B-catalyzed demethylation in the promoter region of SNAI1. Their effects have been evidenced in mice models exposed to a high-fat diet, yet which part of the body composition affected should be further assessed to confirm/rule out possible extra-hepatic effects (toxicity, regulation of lipid metabolism in adipose tissues, etc.). This finding aids in the identification of new targets for the prevention against lipogenesis and steatosis. However, to get to know the extent of this pathway being a potential target in ameliorating NAFLD of the general hepatic condition, additional measurements (such as serum levels of markers of liver damage AST, ALT, inflammatory and apoptosis markers in the liver) are needed. Nevertheless, more investigations are necessary to select small molecule activators before clinical application of these molecules-based target approaches.

## Supplementary Information

**Additional file 1: Fig. S1** Schematic of modeling in mice.

**Additional file 2: Fig. S2** Body weight of mice exposed to high-fat diet following different treatment protocols. A, Body weight of mice exposed to standard/high-fat diet, ***p < 0.001 vs. mice on standard diet; B, Food intake of mice; C, Subcutaneous fat weight of mice; D, Body weight of mice exposed to high-fat diet treated with overexpressed ISL1, ***p < 0.001 vs. mice exposed to high-fat diet treated with oe-NC; E, Body weight of mice exposed to high-fat diet treated with overexpressed KDM6B/SNAI1, ***p < 0.001 vs. mice exposed to high-fat diet treated with oe-NC; F, Body weight of mice exposed to high-fat diet treated with overexpressed ISL1/KDM6B in presence/absence of sh-SNAI1; ***p < 0.001 vs. mice exposed to high-fat diet treated with oe-NC + sh-NC, ###*p* < 0.001 vs. mice exposed to high-fat diet treated with oe-ISL1 + sh-NC, &&&*p* < 0.001 vs. mice exposed to high-fat diet treated with oe-KDM6B + sh-NC; n = 8. n = 8 mice in each group.

**Additional file 3: Fig. S3** Experimental images of Oil red O staining and immunohistochemistry. A, Oil red O staining was applied to detect liver lipid accumulation in mice exposed to high-fat diet; B, Immunohistochemistry was used to detect the expression of ISL1 in liver tissues of mice exposed to high-fat diet; C, Oil red O staining was performed to detect lipid accumulation; D, Oil red O staining was applied to detect the effect of overexpressed ISL1 on liver lipid accumulation in mouse model; E, Oil red O staining was performed to detect the effect of ISL1 on lipid accumulation; F, Oil red O staining shows steatosis; G, Oil red O staining was applied to detect the effect of overexpressing KDM6B and SNAI1 on liver lipid accumulation in mouse model; H, Oil red O staining was performed to detect the effect of KDM6B and SNAI1 on lipid accumulation; I, Oil red O staining was applied to detect the effect of overexpressing ISL1/KDM6B and knockdown of SNAI1 on liver lipid accumulation in mouse model; J, Oil red O staining was performed to detect the effect of overexpressing ISL1/KDM6B and knockdown of SNAI1 on lipid accumulation. n = 8 mice in each group.

**Additional file 4: Fig. S4** Western blots. A, Western blot analysis was conducted to detect the expression of ISL1, lipid synthesis- and lipolysis-related genes in liver tissue of mice exposed to high-fat diet; B, Western blot analysis was used to detect the expressions of ISL1, lipid synthesis- and lipolysis-related genes in an in vitro model of lipid accumulation; C, Western blot analysis was used to detect the ISL1 overexpression efficiency in liver tissues of mice exposed to high-fat diet; D, Western blot analysis was conducted to detect the effect of ISL1 on lipid synthesis- and lipolysis-related genes in liver tissues of mice exposed to high-fat diet; E, Western blot analysis was used to detect the effect of ISL1 on the expressions of lipid synthesis- and lipolysis-related genes in an in vitro model of lipid accumulation; F, Expressions of KDM6B and SNAI1 in liver tissues of mice exposed to high-fat diet detected by Western blot analysis; G, Expressions of KDM6B and SNAI1 in an in vitro model of lipid accumulation determined by Western blot analysis; H, Western blot was used to detect the expression of ISL1/KDM6B/SNAI1 in THLE-2 cells after overexpressing/knockdown of ISL1/KDM6B; I, Western blot analysis was used to detect the KDM6B overexpression efficiency in liver tissues of mice exposed to high-fat diet; J, Western blot analysis was used to detect the SNAI1 overexpression efficiency in liver tissues of mice exposed to high-fat diet; K, Western blot analysis was conducted to detect the effect of KDM6B and SNAI1 on lipid synthesis- and lipolysis-related genes in liver tissues of mice exposed to high-fat diet; L, Western blot analysis was used to detect the effect of overexpressed KDM6B and SNAI1 on the expressions of KDM6B, SNAI1, lipid synthesis- and lipolysis-related genes in an in vitro model of lipid accumulation; M, Western blot analysis was applied to detect the effect of overexpressing ISL1/KDM6B and knockdown of SNAI1 on the expression levels of ISL1, KDM6B, and SNAI1 in the liver tissues of mice exposed to high-fat diet; N, Western blot analysis was conducted to detect the effect of overexpressing ISL1/KDM6B and knockdown of SNAI1 on lipid synthesis- and lipolysis-related genes in liver tissues of mice exposed to high-fat diet; O, Western blot analysis was used to detect the effect of overexpressing ISL1/KDM6B and knockdown of SNAI1 on the expressions of ISL1, KDM6B, SNAI1, lipid synthesis- and lipolysis-related genes in the in vitro model of lipid accumulation. Cell experiment was repeated 3 times independently. n = 8 mice in each group.

**Additional file 5: Fig. S5** Effects of ISL1/KDM6B/SNAI1 on lipid droplet accumulation. A, Relative protein expressions of ISL1, KDM6B, and SNAI1 in mice exposed to standard/high-fat diet. B, Oil red O staining in hepG2 cell line. n = 8 mice in each group.

**Additional file 6: Fig. S6** Relative protein expressions of markers of β-oxidation (CPT1A, CPT2, ACADM) and autophagy (ATG5, ATG6, p62, LC3) in the experiments with overexpressed ISL1, KDM6B and SNAI1. Cell experiment was repeated 3 times independently. n = 8 mice in each group.

**Additional file 7: Fig. S7** Serum transaminase and apoptosis markers in mice of each treatment group. A, AST level determined using colorimetry; B, ALT level determined using colorimetry; C, The c-caspase 3 and c-caspase 7 levels detected using Western blot. n = 8 mice in each group.

**Additional file 8: Table S1**. Primer sequences for reverse transcription quantitative polymerase chain reaction.

## Data Availability

The datasets supporting the conclusions of this article are included within the article and its additional files.

## Abbreviations

NAFLD: Non-alcoholic fatty liver disease
ISL1: Islet1
oe-ISL1: Overexpressed ISL-1
KDM6B: Lysine-specific demethylase 6B
TG: Triglycerides
NASH: Non-alcoholic steatohepatitis
JMJD3: Jumonji domain-containing protein 3
TG: Triglyceride
TC: Cholesterol
sh-SNAI1: Short hairpin RNA against SNAI1
NCs: Negative controls
BCA: Bicinchoninic acid
HE: Hematoxylin–eosin
IHC: Immunohistochemistry
HRP: Horseradish peroxidase
DAB: Diaminobenzidine
SDS: Sodium dodecyl sulfate
SCD1: Stearoyl-CoA desaturase 1
ACC1: Acetyl-CoA carboxylase 1
SREBP1: Sterol-regulatory element binding protein 1
ATGL: Adipose triglyceride lipase
MGL: Monoacylglycerol lipase
HSL: Hormone-sensitive lipase
RT-qPCR: Reverse transcription quantitative polymerase chain reaction
cDNA: Complementary DNA
Co-IP: Co-immunoprecipitation
ChIP: Chromatin immunoprecipitation assay
HE: Hematoxylin–eosin
