# Supplementary material for: Synergistic effects of ISL1 and KDM6B on non-alcoholic fatty liver disease through the regulation of SNAI1
Source: Mol Med. 2022 Jan 31;28:12. doi: 10.1186/s10020-021-00428-7 (PMC8802528; doi:10.1186/s10020-021-00428-7)
Supplement: Supplementary file 8 — Additional file 8: Table S1. Primer sequences for reverse transcription quantitative polymerase chain reaction. [file 10020_2021_428_MOESM8_ESM.docx]

**Table S1** Primer sequences for reverse transcription quantitative polymerase chain reaction

| Gene | Sequence (5' - 3') |
| --- | --- |
| Human β-actin | Forward ACTGGAACGGTGAAGGTGAC |
|  | Reverse AGAGAAGTGGGGTGGCTTTT |
| Human ISL1 | Forward CTGCTTTTCAGCAACTGGTCA |
|  | Reverse TAGGACTGGCTACCATGCTGT |
| Human KDM6B | Forward GCCTCTTCTCCACCAAGACC |
|  | Reverse GCCTGGTACTGTGCGTACTT |
| Human SNAI1 | Forward GCTGCAGGACTCTAATCCAGA |
|  | Reverse ATCTCCGGAGGTGGGATG |
| Mouse β-actin | Forward CCACACCCGCCACCAGTTCG |
|  | Reverse TACAGCCCGGGGAGCATCGT |
| Mouse ISL1 | Forward CTGCTTTTCAGCAACTGGTCA |
|  | Reverse AGGACTGGCTACCATGCTGT |
| Mouse KDM6B | Forward TGAAGAACGTCAAGTCCATTGTG |
|  | Reverse TCCCGCTGTACCTGACAGT |
| Mouse SNAI1 | Forward CACCCTCATCTGGGACTCTC |
|  | Reverse GAGCTTTTGCCACTGTCCTC |

Note: ISL1, islet1; KDM6B, lysine-specific demethylase 6B.
